# Healthcare experiences of LGBTQ+ people: non-binary people remain unaffirmed

**DOI:** 10.3389/fsoc.2024.1448821

**Published:** 2024-08-23

**Authors:** Dustin Z. Nowaskie, Olwen Menez

**Affiliations:** ^1^Department of Psychiatry and the Behavioral Sciences, Keck School of Medicine of University of Southern California, Los Angeles, CA, United States; ^2^Indiana University School of Medicine, Evansville, IN, United States

**Keywords:** discrimination, healthcare, LGBTQ+, non-binary, stigma, transgender

## Abstract

**Introduction:**

Lesbian, gay, bisexual, transgender, queer, and all sexually and gender diverse (LGBTQ+) people experience discrimination across many contexts, including healthcare environments. While some research has shown transgender people and non-binary people often endure higher rates of marginalization than cisgender, sexually diverse people, past data are limited.

**Methods:**

A sample of LGBTQ+ people (*N* = 173) in the United States completed an anonymous, online, self-reported survey, which included the Consumer Assessment of Healthcare Providers and Systems and healthcare experience questions. Groups, including people who identified as cisgender, sexually diverse (*n* = 116), transgender (*n* = 24), and non-binary (*n* = 33), were compared using chi-square and multivariate analysis of covariance tests.

**Results:**

Compared to cisgender, sexually diverse people, non-binary people were less likely to report feeling comfortable with a physical exam, having good mental health, respected by providers, that providers had adequate medical information, that providers could care for someone going through gender affirmation, and that hospital staff were comfortable interacting with them. Additionally, non-binary people were more likely to report hospital staff misgendering them.

**Discussion:**

These unique LGBTQ+ subgroup differences may be secondary to identity-specific stigma that non-binary people face. More international studies are needed to elucidate these subgroup-specific healthcare experiences across LGBTQ+ identities.

## Introduction

It has been well researched that many LGBTQ+ people face higher rates of violence compared to cisgender, heterosexual people (Antebi-Gruszka and Scheer, 2021), which often arise from societal stigmas (Saewyc et al., 2006; Lopez-Saez et al., 2020). LGBTQ+ marginalizations are also found within healthcare settings (Macapagal et al., 2016). Based on minority stress models, LGBTQ+ individuals can encounter heteronormative and cisnormative societal pressures and hostile external environments, which can then lead to internalization and self-stigma (Meyer, 2003; Lopez-Saez et al., 2020). As a result of continued external and internal stigmas, LGBTQ+ individuals may experience significant health disparities over time (Lopez-Saez et al., 2020; Hoy-Ellis, 2023).

Particular subgroups within LGBTQ+ populations, especially gender diverse communities, face greater challenges (Baldwin et al., 2018; Lefevor et al., 2019; Scandurra et al., 2019). For example, transgender individuals find that their greatest barrier to healthcare access is the lack of providers that have sufficient knowledge regarding transgender topics (Safer et al., 2016). Half of all transgender patients report having to teach their medical providers about transgender care (Grant et al., 2011). For gender diverse people, experiencing rejection in medical environments is a frequent and salient internal stressor (Hastings et al., 2021). Sometimes, direct harm is done. In providers’ office and hospitals, 24% of transgender patients report being denied equal treatment, 25% experience harassment or disrespect, and at least 2% report being physically assaulted in healthcare settings (Grant et al., 2011). Furthermore, LGBTQ+ people who have marginalized intersecting identities, such as individuals who identify as multiracial, are sometimes more likely to anticipate and experience discrimination (Alizaga et al., 2022). Transgender patients of Color specifically who have experienced healthcare interactions in which providers responded negatively to their race/ethnicity and/or gender identity state that they believe they would be better treated if they were cisgender or White (Howard et al., 2019).

Within LGBTQ+ communities, gender diverse subgroups have been studied, yet data are often from small, geographically bound areas. For instance, studies assessing health differences in non-binary/genderqueer individuals and transgender individuals have found mixed results (Scandurra et al., 2019). Some studies have shown contrasting differences in health outcomes, including higher rates of victimization for transgender people (Kattari et al., 2021), higher rates of harassment, abuse, and trauma for genderqueer people (Lefevor et al., 2019), and worse mental health for transgender and non-binary people assigned male at birth (Newcomb et al., 2020; Pharr, 2021) or for transgender and non-binary people assigned female at birth (Price-Feeney et al., 2020). Another study found there were no differences in health factors and outcomes between transgender and non-binary/genderqueer people (Nowaskie et al., 2023). What is clear is that more research needs to be conducted on larger, international scales.

Gender diverse people face distinct challenges in healthcare settings (Nowaskie et al., 2023). Many non-binary people specifically experience additional stigma and burden to conform to binary healthcare systems. These challenges range from feeling frustrated and disrespected due to receiving care via binary lenses (Lykens et al., 2018) to receiving less access to affirming medical care (Todd et al., 2019). As a result of societal non-affirmation (Lane et al., 2022) and negative healthcare experiences like misgendering, invalidation, and even pathologization, non-binary people often conceal their identities, erroneously identify with binary terms and labels (Lykens et al., 2018), and avoid obtaining healthcare altogether (Bindman et al., 2022). As such, despite their increasing number and visibility, non-binary people often continue to feel invalidated and unaffirmed in their identities and healthcare experiences (Richards et al., 2016; Scandurra et al., 2019).

The primary purpose of this study was to comprehensively examine LGBTQ+ subgroup healthcare experiences across the United States (U.S.). Aims included exploring experiences with providers, staff, and healthcare systems in general. Based on current limited research demonstrating that LGBTQ+ subgroup differences in discrimination and healthcare disparities exist, it was hypothesized that both transgender people and non-binary people would report more negative experiences compared to cisgender, sexually diverse people.

## Materials and methods

### Design, participants, and variables

This study was deemed exempt by the Indiana University Institutional Review Board (Protocol #11442). Between September and November 2021, an anonymous, self-report, cross-sectional survey was distributed online by a national nonprofit LGBTQ+ health equity organization, OutCare Health (Nowaskie, 2021), via social and marketing channels to LGBTQ+ people across the U.S., to understand the unique healthcare experiences of LGBTQ+ people and promote health equity practices and policies. The survey contained demographics (i.e., age, education, ethnicity, gender identity, insurance type, race, region, and sexual orientation), questions from the Consumer Assessment of Healthcare Providers and Systems Clinician & Group Survey Adult Version Survey 4.0 (beta), which asks participants about their healthcare experiences during their most recent provider visit, and independently constructed items pertaining to general healthcare experiences.

### Analyses

All analyses were conducted on SPSS Statistics 28. Participants who identified as exclusively cisgender and heterosexual (*n* = 6) were removed from the analyses. Remaining LGBTQ+ participants were then sorted into three subgroups based on gender identity: cisgender, sexually diverse (i.e., people who identified as exclusively cisgender men or cisgender women), transgender (i.e., people who identified exclusively as transgender men or transgender women), and non-binary (i.e., people who did not identify with binary terms). Demographic means and frequencies were computed; demographic differences across the subgroups were calculated using chi-square tests. For healthcare experience questions, frequencies, chi-square tests, and multivariate analyses of covariance (MANCOVAs) were examined. MANCOVAs were tested across the subgroups, with demographics as independent variables and covariates and healthcare experience questions as dependent variables. *Post hoc* tests comparing estimated marginal means were used to determine differences between the subgroups. Statistical significance was set at a = 0.05. Given the multitude of survey variables, particular emphases were given to results when the *p*-value was less than 0.01.

## Results

### Demographics

Of the 173 LGBTQ+ people who fully completed the survey, the majority were between the ages of 30 to 50 (*n* = 98, 56.6%, age range: 20 to 81), had at least a college degree (*n* = 140, 80.9%), lived in all U.S. regions, had employer-based insurance (*n* = 125, 72.3%), and identified as exclusively cisgender (*n* = 116, 67.1%); gay, heterosexual, or lesbian (*n* = 89, 51.4%); White or Caucasian (*n* = 147, 85.0%); and not Hispanic and/or Latino/a/x (*n* = 159, 91.9%) (Table 1). Across the subgroups, there were significant differences in: (1) sexual orientation, $x^{2}$ (2) = 22.663, *p* < 0.001, such that more cisgender, sexually diverse people identified with monosexual terms (e.g., gay or lesbian) than transgender people and non-binary people; and (2) race, $x^{2}$ (2) = 10.660, *p* < 0.001, such that more cisgender, sexually diverse people identified as exclusively White or Caucasian than transgender people and non-binary people. There were no significant differences in age, ethnicity, education, region, and insurance type across the subgroups.

**Table 1:** Demographics.

	Cisgender, sexually diverse people (*n* = 116)	Transgender people (*n* = 24)	Non-binary people (*n* = 33)
Age	42.8 (12.5)	44.2 (16.9)	38.6 (12.3)
Sexual orientation			
Asexual	1 (0.9)	–	–
Bisexual	13 (11.2)	2 (8.3)	4 (12.1)
Gay	52 (44.8)	1 (4.2)	3 (9.1)
Heterosexual	–	4 (16.7)	–
Lesbian	20 (17.2)	7 (29.2)	2 (6.1)
Pansexual	7 (6.0)	2 (8.3)	3 (9.1)
Queer	9 (7.8)	3 (12.5)	11 (33.3)
Other	14 (12.1)	5 (20.8)	10 (30.3)
Race			
Prefer not to disclose	3 (2.6)	1 (4.2)	–
Asian or Asian American	–	–	2 (6.1)
Black or African American	1 (0.9)	1 (4.2)	3 (9.1)
White or Caucasian	105 (90.5)	17 (70.8)	25 (75.8)
Other	7 (6.0)	5 (20.8)	3 (9.1)
Ethnicity			
Prefer not to disclose	2 (1.7)	2 (8.3)	–
Yes, Hispanic and/or Latino/a/x	7 (6.0)	–	2 (6.1)
No, not Hispanic and/or Latino/a/x	107 (92.2)	22 (91.7)	30 (90.9)
Education			
Some high school but did not graduate	–	–	1 (3.0)
High school graduate or GED	3 (2.6)	3 (12.5)	–
Some college or 2-year degree	18 (15.5)	3 (12.5)	5 (15.2)
4-year college graduate	21 (18.1)	7 (29.2)	8 (24.2)
More than 4-year college degree	74 (63.8)	11 (45.8)	19 (57.6)
Region			
Midwest	51 (44.0)	12 (50.0)	13 (39.4)
Northeast	10 (8.6)	2 (8.3)	3 (9.1)
South	24 (20.7)	4 (16.7)	7 (21.2)
West	20 (17.2)	2 (8.3)	7 (21.2)
Insurance type			
None/self-pay	6 (5.2)	1 (4.2)	2 (6.1)
Government-based	19 (16.4)	6 (25.0)	7 (21.2)
Employer-based	87 (75.0)	16 (66.7)	22 (66.7)
Other	3 (2.6)	–	2 (6.1)

All variables are *n* (%) or *M* (*SD*). For “other” categories”. Race: (1) Cisgender, sexually diverse: Mestizo, *n* = 1; mixed, *n* = 2; Native Hawaiian or Pacific Islander, *n* = 1; other, *n* = 1; White or Caucasian and Black or African American, *n* = 2. (2) Transgender: other, *n* = 1; White or Caucasian and American Indian or Alaska Native, *n* = 1; White or Caucasian and prefer not to disclose, *n* = 2; White or Caucasian and Sephardic Jewish, *n* = 1. (3) Non-binary: Brown Latinx, *n* = 1; White or Caucasian and Asian or Asian American, *n* = 1; White or Caucasian and other, *n* = 1.

Sexual orientation: (1) Cisgender, sexually diverse: asexual and lesbian and queer, *n* = 1; bisexual and lesbian and queer, *n* = 1; bisexual and pansexual, *n* = 2; bisexual and pansexual and queer, *n* = 3; gay and queer, *n* = 2; lesbian and queer, *n* = 3; pansexual and queer, *n* = 2. (2) Transgender: asexual and bambi lesbian and bisexual and lesbian and pansexual, *n* = 1; bisexual and queer, *n* = 1; gay and queer, *n* = 1; not a high priority/not active, *n* = 1; trixensexual, *n* = 1. (3) Non-binary: bisexual and pansexual and queer, *n* = 2; bisexual and queer, *n* = 3; gay and queer, *n* = 1; lesbian and pansexual, *n* = 1; lesbian and queer, *n* = 1; pansexual and queer, *n* = 2.

### Healthcare experiences

In general, transgender people and non-binary people reported poorer healthcare experiences than cisgender, sexually diverse people (Table 2). In all cases where there were significant differences across the subgroups, non-binary people were significantly more likely to report poorer healthcare experiences than cisgender, sexually diverse people. In almost all these significant cases, non-binary people also reported poorer healthcare experiences than transgender people, although statistical significance was often not apparent, beyond a few exceptions. For instance, compared to cisgender, sexually diverse people, significantly less non-binary people reported: (1) feeling that their provider had the medical information they needed (56.7% vs. 77.5%); (2) trusting the healthcare system (9.4% vs. 43.0%); (3) feeling comfortable with a physical exam (30.3% vs. 63.6%); and feeling that hospital staff (4) would be able to care for someone going through gender affirmation (3.1% vs. 20.9%) and (5) were comfortable when interacting with them (48.4% vs. 81.1%). Likewise, compared to cisgender, sexually diverse people, significantly more non-binary people reported that hospital staff: (1) denied them healthcare (20.8% vs. 5.4%); (2) referred them elsewhere because of their gender (12.0% vs. 2.9%); and (3) misgendered them (38.7% vs. 1.1%).

**Table 2:** Gender-specific differences in healthcare experiences.

	No (%)			Yes, somewhat (%)			Yes, definitely (%)			Chi-square		MANCOVA		
	CS	T	NB	CS	T	NB	CS	T	NB	$x^{2}(4)$	*p*-value	*F*(2, 25)	*p*-value	partial $\eta^{2}$
During your most recent visit, did this provider explain things in a way that was easy to understand?	2.0	8.7	0	12.7	21.7	26.7	85.3	69.6	73.3					
During your most recent visit, did this provider listen carefully to you?	1.0	4.3	0	15.7	21.7	30.0	83.3	73.9	70.0					
During your most recent visit, did this provider show respect for what you had to say?	0	4.3	6.7	10.8	21.7	23.3	89.2	73.9	70.0	10.772	0.029	4.783, CS/NB	0.017	0.277
During your most recent visit, did this provider spend enough time with you?	3.9	13.0	0	14.7	8.7	23.3	81.4	78.3	76.7					
During your most recent visit, did this provider have the medical information they needed about you?	2.0	8.7	0	20.6	13.0	43.3	77.5	78.3	56.7	12.052	0.017	6.340, CS/NB	0.006	0.337
Thinking about your most recent visit, was the staff from this provider’s office as helpful as you thought they should be?	1.2	13.6	15.4	27.2	22.7	34.6	71.6	63.6	50.0	10.780	0.029			
Thinking about your most recent visit, did the staff from this provider’s office treat you with courtesy and respect?	0	4.5	3.8	12.3	18.2	30.8	87.7	77.3	65.4					
	0–4			5			6–10							
Using any number from 0 to 10, where 0 is the worst visit possible and 10 is the best visit possible, what number would you use to rate your most recent visit?	2.0	4.3	0	2.0	8.7	3.3	96.1	87.0	96.7					
	**Poor**			**Fair**			**Excellent, very good, or good**							
In general, how would you rate your overall health?	–	–	–	9.8	25.0	18.2	90.2	75.0	81.8					
In general, how would you rate your overall mental or emotional health?	3.3	16.7	12.1	18.9	20.8	27.3	77.9	62.5	60.6	9.481	0.050	3.420, CS/NB	0.049	0.215
	**Strongly disagree or disagree (%)**			**Neither agree nor disagree (%)**			**Strongly agree or agree (%)**							
The cost of healthcare has affected me.	18.3	16.7	3.1	15.8	8.3	9.4	65.8	75.0	87.5					
Transportation has been a burden for my healthcare.	84.0	65.0	69.7	11.8	15.0	12.1	4.2	20.0	18.2	10.477	0.033			
Finding child/elder care has been a burden for my healthcare.	84.1	64.3	47.4	7.3	14.3	21.1	8.5	21.4	31.6	12.580	0.014			
Cultural barriers have affected my healthcare.	70.3	52.4	40.0	13.5	19.0	20.0	16.2	28.6	40.0	11.153	0.025			
Fear, depression, anxiety, and/or stress have held me back from scheduling a healthcare visit.	51.7	39.1	19.4	10.0	8.7	9.7	38.3	52.2	71.0	11.931	0.018			
The thought of missing work has affected my healthcare.	43.2	42.1	26.7	11.9	15.8	20.0	44.9	42.1	53.3					
Hospital staff have denied me healthcare.	88.3	52.4	70.8	6.3	28.6	8.3	5.4	19.0	20.8	19.449	<0.001			
Hospital staff have referred me somewhere else because of my gender.	92.4	65.0	72.0	4.8	15.0	16.0	2.9	20.0	12.0	15.388	0.004			
It is difficult for me to schedule a healthcare appointment.	61.3	50.0	46.9	18.5	25.0	12.5	20.2	25.0	40.6					
It is difficult for me to have health insurance.	71.4	58.3	64.5	14.3	25.0	6.5	14.3	16.7	29.0					
Hospital staff misgendered me.	96.8	56.5	45.2	2.2	13.0	16.1	1.1	30.4	38.7	47.724	<0.001	59.633, CS/NB, T/NB	<0.001	0.827
Hospital staff asked inappropriate questions about my gender.	88.2	59.1	78.6	5.4	9.1	14.3	6.5	31.8	7.1	15.456	0.004			
Hospital staff viewed my gender as a disease.	93.5	72.7	76.9	5.4	22.7	23.1	1.1	4.5	0	11.639	0.020			
Hospital staff were discriminatory.	76.6	69.6	67.7	16.8	26.1	22.6	6.5	4.3	9.7					
It is easy to find appropriate healthcare.	45.0	50.0	78.8	16.7	12.5	12.1	38.3	37.5	9.1	13.138	0.011			
I am willing to participate in healthcare decisions.	0.8	4.2	0	0.8	4.2	0	98.3	91.7	100					
I trust the healthcare system.	33.9	41.7	46.9	23.1	16.7	43.8	43.0	41.7	9.4	14.318	0.006			
I am confident that hospital staff would be able to care for someone going through gender affirmation.	53.9	37.5	81.3	25.2	16.7	15.6	20.9	45.8	3.1	18.429	0.001	5.085, CS/NB, T/NB	0.014	0.289
Gender influences my healthcare.	35.4	4.2	9.4	15.9	8.3	12.5	48.7	87.5	78.1	19.730	<0.001			
I am satisfied with wait times for healthcare visits.	44.2	43.5	43.8	23.3	13.0	28.1	32.5	43.5	28.1					
I am comfortable with a physical exam.	11.9	29.2	51.5	24.6	25.0	18.2	63.6	45.8	30.3	25.474	<0.001	7.049, CS/T, CS/NB	0.004	0.361
I am satisfied with the healthcare system.	52.5	58.3	81.8	20.0	25.0	9.1	27.5	16.7	9.1	10.355	0.035			
The patient form was inclusive.	33.3	34.8	53.3	22.5	17.4	10.0	44.1	47.8	36.7			3.597, CS/NB	0.042	0.223
My medical information was incorrect.	66.4	56.5	53.1	8.8	13.0	15.6	24.8	30.4	31.3					
Hospital staff addressed me by the name I go by.	3.7	4.3	12.5	2.8	4.3	6.3	93.6	91.3	81.3			4.277, CS/NB	0.025	0.255
Hospital staff were comfortable when interacting with me.	9.4	8.7	9.7	9.4	43.5	41.9	81.1	47.8	48.4	24.662	<0.001	4.842, CS/NB	0.017	0.279
Hospital staff were coordinated.	16.3	13.0	13.3	16.3	30.4	30.0	67.3	56.5	56.7					

CS, cisgender, sexually diverse people; MANCOVA, multivariate analysis of covariance; NB, non-binary people; T, transgender people.Controls in MANCOVA analyses were age, education, ethnicity, insurance type, race, region, and sexual orientation (omitted from table).Slashes denote significant subgroup comparisons, with the first subgroup as the reference group.

Compared to cisgender, sexually diverse people, significantly less transgender people reported feeling: (1) comfortable with a physical exam (45.8% vs. 63.6%); and (2) that hospital staff were comfortable when interacting with them (47.8% vs. 81.1%). Additionally, significantly more transgender people reported that hospital staff: (1) denied them healthcare (19.0% vs. 5.4%), (2) referred them elsewhere because of their gender (20.0% vs. 2.9%), and (3) misgendered them (30.4% vs. 1.1%).

## Discussion

This is the first known study to comprehensively examine LGBTQ+ healthcare experiences nationally within provider, staff, and healthcare system contexts and across LGBTQ+ subgroups with cisgender, sexually diverse, transgender, and non-binary representations. Overall, LGBTQ+ people reported high rates of healthcare discrimination. As past research has indicated (Franks et al., 2023; Jackson et al., 2023; Bromdal et al., 2024), gender diverse people conveyed much higher rates of stigma and cultural, clinical, and structural barriers. Interestingly, in some contexts, non-binary people reported higher rates of marginalization than transgender people.

In general, LGBTQ+ people reported negative experiences with provider interactions. Some LGBTQ+ people noted providers not having enough information, listening carefully, showing respect, explaining information well, and many more conveyed providers not spending enough time with them. While many of these shortcomings were shared experiences across cisgender, sexually diverse people, transgender people, and non-binary people, more non-binary people were less likely to report feeling respected by providers and that providers had adequate medical information compared to cisgender, sexually diverse people. As previous research has shown (Nowaskie et al., 2019), LGBTQ+ people reported more negative experiences with staff, such as not feeling helped or respected, than with providers. Given the high turnover in particular staff occupations (e.g., front desk employees), staff members may be less likely to engage in LGBTQ+ education and training and therefore more likely to harbor and externalize biases and discrimination. Future research should consider the varying influence of various healthcare employees, i.e., providers and staff, on perceptions of care and health for LGBTQ+ people. Such employee-specific analyses would likely yield insightful gaps and tailored LGBTQ+ training, e.g., clinical disparities and treatment topics for providers and affirming communication techniques for frontline staff.

Additionally, LGBTQ+ people reported many negative experiences with healthcare systems. Some LGBTQ+ people expressed burdens of care such as transportation, finding child or elder support, cultural factors, scheduling appointments, and having insurance. Many more LGBTQ+ people conveyed barriers of care including cost, mental distress, missing work, finding appropriate healthcare, and wait times for healthcare visits. Likewise, LGBTQ+ people disclosed that hospital staff participated in outright denying healthcare, referring elsewhere, misgendering, asking inappropriate questions, as well as being discriminatory, uncomfortable, and uncoordinated. Such disparities likely directly contributed to LGBTQ+ people asserting not feeling comfortable with physical exams, distrusting and feeling dissatisfied with healthcare systems, and not feeling confident that healthcare systems can care for people undergoing gender affirmation. This marginalization was much more prominent in non-binary people.

Similar to previous studies documenting that healthcare disparities exist and vary across LGBTQ+ subgroups (Lefevor et al., 2019; Scandurra et al., 2019; Philips et al., 2024), a moderate amount of LGBTQ+ people in this study, especially transgender people and non-binary people, stated that their overall health was only fair and that their mental health was poor. Healthcare discrimination from providers, staff, and systems likely perpetuates stigma, leading to chronic stress and contributing to these healthcare disparities. While LGBTQ+ stigma exists across many various healthcare contexts and should be addressed systemically, future research should consider the variable impact of burdens of care on LGBTQ+ communities as a whole and across LGBTQ+ subgroups.

Non-binary people remain unaffirmed. Across multiple healthcare contexts, non-binary people reported marginalization from staff and systems. These LGBTQ+ subgroup-specific differences are likely secondary to the burden of invalidating binary healthcare systems that non-binary people face much more so than cisgender, sexually diverse people and perhaps even transgender people (Richards et al., 2016; Lykens et al., 2018; Scandurra et al., 2019). While this data contributes to an understanding of LGBTQ+ non-affirmation, it is a mere snapshot of the entire extensive healthcare industry and does not account for many more potential areas of discrimination within specific types of staff, providers, leadership, groups, departments, and communities. It also does not fully consider social and political contexts. For instance, these disparities are plausibly a function of the current socio-politico-medico systemic and structural stigmas, including overt anti-LGBTQ+ bans and legislations that limit and even prevent public visibility, legal recognition, and access to gender affirming care, that gender diverse people continue to endure (Jackson et al., 2023; Bromdal et al., 2024). Inspections into these many areas are absolutely needed.

### Limitations

There are study limitations to note. While these data represent experiences from a national sample, the sample size should be viewed as a pilot. Ideally, LGBTQ+ healthcare experiences should be re-examined with larger, more generalizable national samples that represent more racially and ethnically diverse LGBTQ+ people. Samples should also be more equal in distribution. Given the online nature of data collection, a response rate was not calculable, LGBTQ+ healthcare experiences may have been under-or overreported, and data may not be generalizable to LGBTQ+ individuals with barriers to or entirely without online technology and accessibility. Additionally, individuals with more negative experiences may have been more inclined to respond to the survey. A control group with cisgender, heterosexual people was also not undertaken; while it is likely that cisgender, heterosexual people have less negative healthcare experience than LGBTQ+ people, exact data are unknown. Moreover, this study examined cisgender, sexually diverse people in a homogenous manner and did not assess for subgroup differences with this group (e.g., gay and lesbian people compared to bisexual and pansexual people). While this study also highlighted understudied non-binary populations in a homogenous manner, this subgroup in itself often represents many identities (e.g., agender, genderfluid, genderqueer, gender nonconforming, and non-binary) which may or may not have unique healthcare experiences. Larger samples with each of these identities are necessary to explain potential differences.

## Conclusion

LGBTQ+ communities suffer from high rates of healthcare discrimination. There appears to be subtle, yet quite significant, differences between LGBTQ+ subgroups in their healthcare experiences, with non-binary people experiencing much more marginalization than cisgender, sexually diverse people and transgender people. These unique differences may be secondary to identity-specific stigmas that non-binary people face. Much more international data are necessary to elucidate these subgroup-specific healthcare experiences and, more importantly, elevate non-binary and gender diverse perspectives and voices, initiate clinical guidelines, precipitate social policies, institute socio-politico-medico systemic and structural changes, and, above all, improve care, health, and well-being.

## Data Availability

The original contributions presented in the study are included in the article/supplementary material, further inquiries can be directed to the corresponding author.
